# Post-stroke dementia – a comprehensive review

**DOI:** 10.1186/s12916-017-0779-7

**Published:** 2017-01-18

**Authors:** Milija D. Mijajlović, Aleksandra Pavlović, Michael Brainin, Wolf-Dieter Heiss, Terence J. Quinn, Hege B. Ihle-Hansen, Dirk M. Hermann, Einor Ben Assayag, Edo Richard, Alexander Thiel, Efrat Kliper, Yong-Il Shin, Yun-Hee Kim, SeongHye Choi, San Jung, Yeong-Bae Lee, Osman Sinanović, Deborah A. Levine, Ilana Schlesinger, Gillian Mead, Vuk Milošević, Didier Leys, Guri Hagberg, Marie Helene Ursin, Yvonne Teuschl, Semyon Prokopenko, Elena Mozheyko, Anna Bezdenezhnykh, Karl Matz, Vuk Aleksić, DafinFior Muresanu, Amos D. Korczyn, Natan M. Bornstein

**Affiliations:** 1 Neurology Clinic, Clinical Center of Serbia, School of Medicine, University of Belgrade, Dr Subotica 6, 11000 Belgrade, Serbia; 2 Department of Clinical Neurosciences and Preventive Medicine, Danube University Krems, Krems, Austria; 3 Max Planck Institute for Metabolism Research, Cologne, Germany; 4 Institute of Cardiovascular and Medical Sciences, University of Glasgow, Glasgow, UK; 5 Department of internal medicine, Oslo University Hospital, Ullevål and Department of Medical Research, Bærum Hospital, Vestre Viken Hospital Trust, Bærum, Norway; 6 Department of Neurology, University Hospital Essen, Essen, Germany; 7 Stroke Unit, Department of Neurology, Tel-Aviv Sorasky Medical Center, Tel-Aviv, Israel; 8 Shaare Zedek Medical Center, Jerusalem, Israel; 9 Department of Neurology, Radboud University Medical Center, Donders Institute for Brain, Cognition and Behaviour, Nijmegen, The Netherlands; 10 Department of Neurology and Neurosurgery, McGill University at SMBD Jewish General Hospital and Lady Davis Institute for Medical Research, Montreal, Québec Canada; 11 Department of Rehabilitation Medicine, Pusan National University School of Medicine, Busan, Republic of Korea; 12 Department of Physical and Rehabilitation Medicine, Sungkyunkwan University School of Medicine, Center for Prevention and Rehabilitation, Heart Vascular and Stroke Institute, Samsung Medical Center, Seoul, Republic of Korea; 13 Department of Neurology, Inha University School of Medicine, Incheon, South Korea; 14 Hallym University Medical Center, Kang Nam Sacred Heart Hospital, Seoul, South Korea; 15 Department of Neurology, Gachon University Gil Medical Center, Incheon, South Korea; 16 Department of Neurology, University Clinical Center Tuzla, School of Medicine University of Tuzla, 75000 Tuzla, Bosnia and Herzegovina; 17 Department of Internal Medicine, University of Michigan and the VA Ann Arbor Healthcare System, Ann Arbor, MI USA; 18 Department of Neurology, Rambam Health Care Campus, Haifa, Israel; 19 Technion Faculty of Medicine, Haifa, Israel; 20 Centre for Clinical Brain Sciences, University of Edinburgh, Edinburgh, UK; 21 Clinic of Neurology, Clinical Center Nis, Nis, Serbia; 22 U1171-Department of Neurology, University of Lille, Inserm, Faculty of Medicine, Lille University Hospital, Lille, France; 23 Department of Neurology and Medical Rehabilitation, Krasnoyarsk State Medical University named after Professor V.F. Voyno-Yasenetsky, Krasnoyarsk, Russia; 24 Department of Neurosurgery, Clinical Hospital CenterZemun, Belgrade, Serbia; 25 Department of Clinical Neurosciences, “Iuliu Hatieganu” University of Medicine, Clij-Napoca, Romania; 26 Department of Neurology, Tel Aviv University, Ramat Aviv, 69978 Israel

**Keywords:** Cognitive impairment, Dementia, Definitions and classification, Diagnosis, Neuroimaging, Interventions, Biomarkers, Stroke

## Abstract

Post-stroke dementia (PSD) or post-stroke cognitive impairment (PSCI) may affect up to one third of stroke survivors. Various definitions of PSCI and PSD have been described. We propose PSD as a label for any dementia following stroke in temporal relation. Various tools are available to screen and assess cognition, with few PSD-specific instruments. Choice will depend on purpose of assessment, with differing instruments needed for brief screening (e.g., Montreal Cognitive Assessment) or diagnostic formulation (e.g., NINDS VCI battery). A comprehensive evaluation should include assessment of pre-stroke cognition (e.g., using Informant Questionnaire for Cognitive Decline in the Elderly), mood (e.g., using Hospital Anxiety and Depression Scale), and functional consequences of cognitive impairments (e.g., using modified Rankin Scale). A large number of biomarkers for PSD, including indicators for genetic polymorphisms, biomarkers in the cerebrospinal fluid and in the serum, inflammatory mediators, and peripheral microRNA profiles have been proposed. Currently, no specific biomarkers have been proven to robustly discriminate vulnerable patients (‘at risk brains’) from those with better prognosis or to discriminate Alzheimer’s disease dementia from PSD. Further, neuroimaging is an important diagnostic tool in PSD. The role of computerized tomography is limited to demonstrating type and location of the underlying primary lesion and indicating atrophy and severe white matter changes. Magnetic resonance imaging is the key neuroimaging modality and has high sensitivity and specificity for detecting pathological changes, including small vessel disease. Advanced multi-modal imaging includes diffusion tensor imaging for fiber tracking, by which changes in networks can be detected. Quantitative imaging of cerebral blood flow and metabolism by positron emission tomography can differentiate between vascular dementia and degenerative dementia and show the interaction between vascular and metabolic changes. Additionally, inflammatory changes after ischemia in the brain can be detected, which may play a role together with amyloid deposition in the development of PSD. Prevention of PSD can be achieved by prevention of stroke. As treatment strategies to inhibit the development and mitigate the course of PSD, lowering of blood pressure, statins, neuroprotective drugs, and anti-inflammatory agents have all been studied without convincing evidence of efficacy. Lifestyle interventions, physical activity, and cognitive training have been recently tested, but large controlled trials are still missing.

## Background

Stroke is a leading cause of disability [1]. Research and interventions have historically focused on physical disabilities [2], while cognitive impairment – an important aspect for stroke survivors – has been rather neglected [3, 4]. Even minor stroke affects daily functioning, executive functions, and cognition, consequently affecting participation, quality of life, and return to work [5]. Stroke survivors are at increased risk of developing cognitive impairment. Obviously, the acute tissue damage may affect cognition. Nevertheless, despite prospective data being available, results are conflicting and the direct cognitive effect of a stroke event beyond the cognitive decline associated with age and vascular risk factors remains poorly understood. Physical impairments tend to improve, to a greater or lesser degree, following stroke; however, for reasons which remain unknown, cognitive impairments progressively worsen.

This review paper is based on the proceedings of the International Congress on Vascular Dementia, Ljubljana, 2015, at which reviews of the literature on post-stroke cognitive impairment (PSCI) and post-stroke dementia (PSD) were presented by leaders of the field and discussed by a broad audience. Key thematic areas were chosen for further elucidation in smaller working groups and, after further discussions, a final review was compiled.

## Methods of research

Using a focused search of PubMed/Medline from January 1995 until August 2016, the relevant literature on PSCI/PSD was critically reviewed. The following keywords were employed in te search: “cognitive impairment”, “dementia”, “definitions and classification”, “diagnosis”, “neuroimaging”, “interventions”, “biomarkers”, and “stroke”. References from the selected papers published in the English language were evaluated and included if they were found to be relevant to the focus of this systematic review. Experts were divided in four groups. The first group of experts conducted research about basic concepts, definitions, and epidemiology of PSD following stroke as well as on tools for assessment of cognitive impairment. The second group evaluated biomarkers for PSD, whereas as neuroimaging studies and interventions for PSD prevention and treatment were critically reviewed by the third and fourth groups, respectively.

## Definitions

A variety of classifications, diagnostic criteria, and descriptive syndromes are used to define PSCI [6], but a widely accepted and harmonized terminology is still missing [7]. Post-stroke neuropsychological syndromes overlap – PSCI is responsible for a substantial number of vascular cognitive impairment (VCI) syndromes; PSCI includes the subgroups of PSD and PSCI not fulfilling criteria for dementia.

The direct application of established diagnostic criteria for dementia may not be suited to stroke populations. For example, the differentiation of dementia from PSD and PSCI not fulfilling criteria for dementia is usually based on limitations in activities of daily living. In stroke survivors with substantial physical impairments it may be difficult to assess changes in activities of daily living related to specifically cognitive problems [8]. Usual definitions of dementia emphasize the presence of multidomain cognitive impairments, and in particular memory deficits. However, in stroke, it may be possible to have disabling cognitive problems but retain memory [9].

One of the main questions in the relationship between stroke and dementia occurring after the cerebrovascular event is whether the stroke causes the cognitive deterioration, contributes to it, or just alerted the physicians to the problem. The question remains to be answered, but undoubtedly about one-third of stroke survivors are found to have a significant degree of cognitive impairment within the first months after the event [10].

We propose the term PSD for any dementia which develops following a clinical cerebrovascular event. Although we use the descriptor “post-stroke”, emerging evidence suggests that transient ischemic attack may also be associated with adverse cognitive prognosis [11]. Using the term PSD in this way does not suggest a particular underlying neuropathological process. This seems appropriate as dementia following stroke often comprises a mix of “vascular” insults and neurodegenerative processes [12]. Stroke occurs predominantly in older adults and therefore stroke patients may have pre-stroke cognitive decline of varying severity [13]. Recognizing the pre-stroke cognitive state is essential to allow appropriate classification, e.g., a patient with pre-existing cognitive impairment (diagnosed or undiagnosed) who then has a minor stroke should not be labeled as PSD.

The time assessment of cognitive impairment is another relevant diagnostic factor. Acute deficiencies in cognitive test scores are often observed following a stroke and retesting after several weeks often reveals improvements [14, 15]. Therefore, the final diagnosis of PSD should be delayed to at least 6 months after the event. We recognize that certain strategic infarcts (for example, in dominant medial temporal lobe) are associated with immediate cognitive syndromes; however, we would still reserve the dementia diagnosis until at least 6 months. This delay in applying a dementia diagnostic label is in keeping with International classification systems such as the American Psychiatric Association Diagnostic and Statistical Manual.

The definition we have proposed is in accordance with other groups. The Vascular Impairment of Cognition Classification Consensus Study (VICCCS; http://www.vicccs.info/) considered PSD as a major subtype of VCI and felt that this label should be used where there is a clear temporal relationship between stroke and cognitive decline. A national Korean study of VCI epidemiology defined PSD as any major cognitive impairment seen at more than 3 months after a stroke event, regardless of pre-stroke cognitive status [16].

**Fig. 1:**
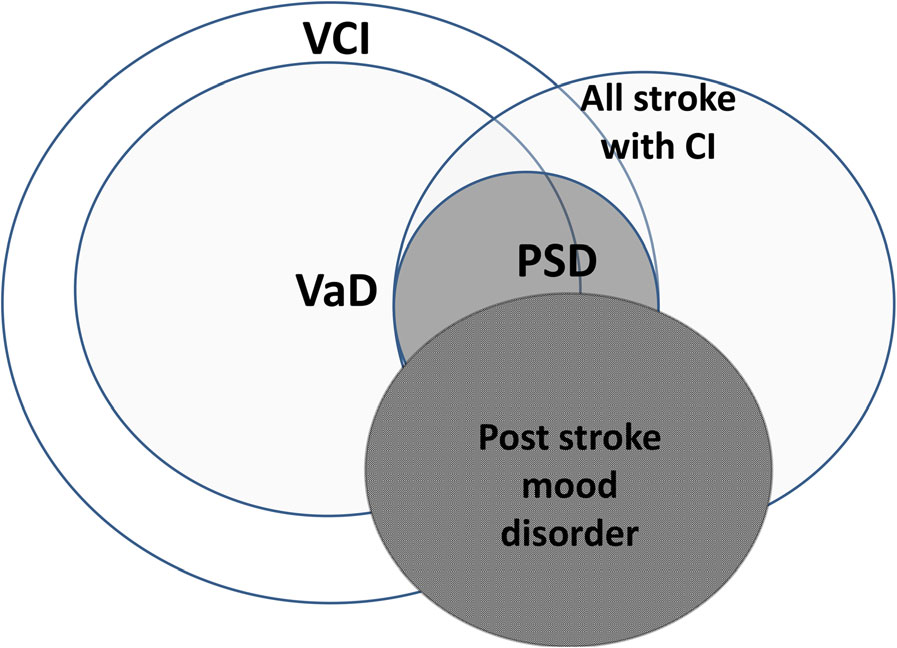
Venn diagram illustrating the overlap of constructs used to define cognitive states relevant to stroke. *CI* cognitive impairment, *PSD* post-stroke dementia, *VaD* vascular dementia, *VCI* vascular cognitive impairment

The terms PSD and vascular dementia (VaD) are not synonymous. The often used term, VaD, has evolved during the last century. Today, VaD represents a concept which includes not only multiple cortical and/or subcortical infarcts, but also strategic single infarcts, non-infarction white matter lesions, hemorrhages, and hypoperfusion as possible causes of dementia. VaD can be considered a subgroup of VCI, representing fully developed dementia after a clearly identified vascular event (the overlap of these definitions is illustrated in Fig. 1).

## Assessments

International guidelines recommend cognitive and mood assessment for all stroke survivors and it is increasingly recognized that cognitive assessment should be part of the “routine” neurological examination in research and clinical practice [17, 18]. Several cognitive assessment tools are available and there is no accepted consensus on preferred approach [19, 20]. Stroke-specific cognitive assessment tools are available [21], but most centers still use tools developed for non-stroke populations. A systematic review of test properties in stroke did not show clear superiority with respect to global accuracy [22]. The most suitable assessment will vary with the purpose of testing; for example, if a rater wishes to ensure all potential cases of PSD are identified then a highly sensitive scale such as the Montreal Cognitive Assessment (MoCA) would be preferable. The cut point used to define PSD can also be adjusted; for example, many centers recommend a lower threshold for MoCA when used in stroke settings. Choice of assessment should also be guided by other factors such as availability, familiarity, and feasibility (Table 1). Feasibility is of particular concern in acute stroke, where severity of disease may preclude lengthy neuropsychological testing [23].

**Table 1:** Properties of selected post-stoke dementia assessment scales

Test	Sensitivity	Specificity	Free to use	Time to administer (min)	Validated in stroke	Suitable for aphasia
ACE-R	0.96	0.70	Yes	20	Yes	No
IQCODE^a^	0.81	0.83	Yes	5	Partial	Yes
MMSE	0.72	0.82	No	5	Yes	No
MoCA (<26/30)	0.95	0.45	Yes	10	Yes	No
MoCA (<22/30)	0.85	0.76	Yes	10	Yes	No
R-CAMCOG	0.81	0.92	No	15	Yes	No

Test properties are from meta-analyses and describe accuracy at conventional thresholds unless otherwise stated

*ACE-R* Addenbrookes Cognitive Evaluation Revised, *MMSE* Mini-Mental State Examination, *MoCA* Montreal Cognitive Assessment, *R-CAMCOG* Rotterdam CAMCOG

^a^Accuracy of IQCODE for assessment of PSD in the longer term after stroke

Informant-based structured questionnaires can capture the patient’s cognitive state before the stroke. The Informant Questionnaire for Cognitive Decline in the Elderly (IQCODE) is the most commonly employed assessment [24]; it has reasonable accuracy in determining dementia, it is available in several languages, and can be completed in minutes using the short form version [25]. IQCODE has been used for assessment of pre-stroke and post-stroke cognition and to aid prognosis; indeed, the properties of IQCODE vary according to the purpose of testing. Testing of cognition should be complemented by functional assessment. The modified Rankin Scale and Barthel Index are the most commonly used functional assessment tools [26]. Post-stroke cognitive issues will often coexist with other neuropsychological problems and should be assessed by validated tools as, for example, language disorders (Frenchay Aphasia Screening Test), mood disorder (Hospital Anxiety Depression Scale), fatigue (Fatigue Severity Scale), delirium (Confusion Assessment Method), and apathy (Apathy Evaluation Scale).

Common stroke-related impairments can complicate assessment if, for example, the patient is unable to complete a pencil and paper task if the dominant hand is weak [27]. Cognitive assessment in the presence of aphasia is particularly problematic. Tools designed for use in aphasia are available such as the Cognitive Assessment Scale for Stroke Patients, which can be administered without using language, and the Oxford Cognitive Screen [27].

## Epidemiology

Stroke is recognized as one of the major causes of adult disability globally. VaD including PSD is the second most common cause of cognitive decline, with only Alzheimer disease (AD) being more prevalent [28]. The lifetime risk of developing either stroke or dementia at age of 65 is one in three in men and one in two in women [29]. With changing population demographics, increased life expectancy and improved survival from stroke, the absolute numbers of patients with PSD will increase. However, due to its relation with stroke incidence, PSD might be reduced with improved stroke prevention.

A pooled analysis of international data classified important contributors and risk factors to PSD as demographic parameters, factors related to pre-stroke functional status and to the index stroke (s), and factors relating to post-stroke complications and brain imaging factors (Table 2) [30, 31].

**Table 2:** Risk factors for PSD [30, 31]

Demographic factors	Age (over 65 years)
	Lower educational level
	Female sex
	Non-Caucasian origin
Pre-stroke factors	Physical impairment
	Cognitive decline
Index stroke factors	Hemorrhagic stroke
	Supratentorial stroke location
	Dominant hemisphere stroke
	Recurrent strokes
Post-stroke factors	Infection
	Delirium
	Early seizures
Neuroimaging factors	Cerebral small-vessel disease
	Cortical atrophy
	Medial temporal lobe atrophy

Silent brain infarctions (SBI) are common findings in elderly patients, but their relationship to dementia remains uncertain. Bornstein et al. [32] studied admission CT scans of 175 consecutive patients without dementia, presenting with a first stroke incident. SBI were defined as CT evidence of stroke not compatible with the acute event. Stroke survivors were followed for their mental state for 5 years and the authors found that SBI did not predict the development of PSD.

According to study of Grau-Olivares and Arboix [33], ischemic cerebral small-vessel disease (SVD) should be regarded as a severe condition prodrome of subcortical VaD, rather than a relatively benign disorder, as considered earlier, especially since SVD can be seen on MRI studies (white matter lesions, lacunes, and microbleeds). Recent studies showed that the proportion of dementia caused by SVD ranges from 36 to 67% [9, 28, 31, 33]. Further, patients with a first-ever lacunar stroke present with cognitive impairment in more than half of cases and more than 55% of patients fulfill mild cognitive impairment criteria [33]. Additionally, the presence of silent multiple lacunar infarctions in patients with first-ever lacunar stroke is an independent predictor of poor performance on executive functions and short delayed verbal memory tests. Thus, in the initial stages of SVD, mild neuropsychological abnormalities appear to be related to lacunes rather than to perivascular hyperintensities of vascular cause or leukoaraiosis [34].

A number of important prospective cohort studies have documented cognitive change following stroke [35, 36]. Meta-analysis of these studies suggests that some form of cognitive impairment affects up to one third of stroke survivors; this figure does not differentiate stroke etiology and may include a proportion (estimated at around 10%) with pre-stroke dementia. Prevalence of PSCI is higher in those with recurrent stroke events [31]. These data might underestimate the true prevalence of post-stroke cognitive change as patients included in studies tend to exclude those with larger strokes who are unable to consent or attend follow-up visits [37]. There are also issues with feasibility of cognitive testing and attrition to follow-up that will contribute to under-estimation of the true burden of cognitive impairment [38].

**Fig. 2:**
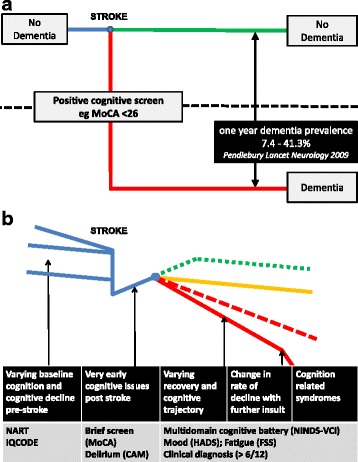
Cognitive “trajectory” in stroke. **a** A traditional view of post-stroke cognitive decline where, following a stroke, some have a degree of fixed cognitive decline causing a dementia that can be detected using a cognitive screening tool. **b** The “real world”, where there are various degrees of pre-stroke cognitive decline and various post-stroke various cognitive trajectories. This complexity requires differing approaches to assessment at various time-points

PSD is often recognized in the first weeks to months post ictus and, thereafter, prevalence of PSD increases with time. Compared to controls with no stroke, incident stroke is associated with acute change in cognition and change in rate of temporal cognitive decline [39]. Pooled analyses of longitudinal cohorts suggest a linear increase of 3 and 1.7% per year in hospital- and population-based studies, respectively [31]. In contrast to cognitive changes in the normal aging population, a linear rate of change is not observed, but stroke survivors seem to show different cognitive “trajectories” and these trajectories may change over time (Fig. 2).

## Biomarkers and predictors of PSD

Biomarkers for PSD may include neuroimaging measures and metabolic, genetic, and inflammatory mediators [40]. Both neurodegenerative and vascular mechanisms are activated and probably result in overlapping processes, therefore sharing some pathological features and likely having some common biomarkers.

### Genetic markers

Genes underlying PSD and VCI may include those shared by VaD and AD, namely genes involved in the pathways of amyloid production or elimination (apolipoprotein E and possibly presenilin and APP) may confer susceptibility to develop dementia after vascular brain injury, thus explaining, at least in part, the well-known interaction and synergism between VaD and AD. Polymorphisms (SNPs) in genes reported as conferring susceptibility to develop PSD and VaD include the e4 allele of apolipoprotein E, a well-known risk factor for AD, although its association with VaD and PSD has been controversial [41–47]. Another reported SNP is in the gene encoding the angiotensin-converting enzyme, one of the enzymes in the renin-angiotensin system. Studies investigating the angiotensin-converting enzyme SNP as a predictor for post-stroke cognitive decline have yielded controversial data [48–52]. A genome-wide association study in the Rotterdam cohort revealed a genetic locus associated with VaD, located on the X chromosome close to the androgen receptor gene [53]. An additional SNP is endothelial nitric oxide synthase, reported as associated with incident dementia in elderly stroke survivors [54]. The main research areas currently being investigated are genetic predisposition, predominantly in genes linked to both AD and vascular disease and inflammatory response.

### Cerebrospinal fluid (CSF) biomarkers

So far, the search for a precise and reliable biochemical indicator which would help establish the diagnosis of VaD and PSD has not been productive. Although, the Aβ-42 peptide and tau protein CSF levels are sensitive markers of AD, they showed the lowest specificity in VaD [55]. Promising results were obtained measuring the levels of matrix-degrading metalloproteinases (MMPs) in CSF, such as gelatinase A (MMP-2) and gelatinase B (MMP-9) [56, 57]. Gelatinases interfere with molecules of the basal lamina, leading to proteolytic disruption of the blood brain barrier (BBB). Further, they are involved in myelin breakdown. Brain ischemia induces a gelatinase A and B elevation at different time points after the stroke. Recent studies showed that the MMP-9 CSF level is significantly higher in VaD patients compared to AD patients as well as elderly healthy subjects, and may help in distinguishing AD from VaD [56]. Other CSF markers also reported as associated with VaD, and indirectly PSD, are levels of α-1 antitrypsin, plasminogen activator inhibitor-1, and apolipoprotein H [58].

### Serum biomarkers

Serum biomarkers, such as β secretase enzyme and receptor levels for advanced glycation end products, have been suggested to correlate with early PSCI [45]. Homocysteine, vitamin B12, and folic acid levels were linked to PSCI; however, supplementation with B vitamins had no effect on the incidence of cognitive impairment or decline [59].

## Inflammatory mediators

Inflammation in stroke seems to have both detrimental and beneficial effects. Activation of resident cells (microglia, astrocytes, and endothelial cells) appears to be neuroprotective and promotes brain regeneration/recovery, whereas recruitment of immune cells with a subsequent increase in the expression of inflammatory mediators, such as reactive oxygen species, cytokines, and chemokines, together with BBB disruption, could lead to edema and neuronal death [60]. Chronic activation of innate immune responses can trigger neurotoxic pathways leading to progressive degeneration. Damaged neurons themselves may also exacerbate immune-mediated disease by release of chemokines and activation of microglia [61]. Several recent longitudinal studies have investigated the relationship between inflammatory markers and PSD, but an association is not yet established. Erythrocyte sedimentation rate (ESR), C-reactive protein, interleukin 6, and interleukin 12 were suggested as predictors of PSCI [62, 63]. Recently, Kliper et al. [64] showed strong relationship between ESR and cognitive performance among stroke survivors, where higher ESR levels were associated with worse performance in cognitive tests, particularly memory scores. Findings from the TABASCO study have raised the possibility that many individuals with cerebrovascular ischemic events harbor a long and persistent pro-inflammatory background [64]. Whether lowering inflammation can also prevent PSD needs to be addressed in prospective clinical trials.

## Peripheral microRNA (miRNA) profiles as novel biomarkers

miRNAs are a type of small non-coding RNA molecules that act as endogenous regulators of gene expression by binding to complementary sequences of target messenger RNA. The dysregulation of miRNAs is thought to be involved in many diseases, and changes in the miRNA expression profile have been observed in cerebral ischemia and AD. Studies have demonstrated that serum miRNAs miR-93, miR-146a, and miR-31 were significantly up regulated in VaD compared to the controls; thus, they could serve as specific biomarkers to discriminate AD from VaD [65]. Endogenous circulating miRNAs have been found to be stable and consistently detected in serum, plasma and other body fluids because of their packaging and secretion into the blood within exosomes. Currently, information regarding miRNAs in VaD and PSD studies is limited, but the potential diagnostic value of miRNAs is very promising. In conclusion, no molecular biomarkers have been confirmed as being specifically associated with PSD.

## Neuroimaging

Neuroimaging in PSD provides significant information about the anatomical substrate of the disorder and has an important role in PSD diagnosis. Further, it adds to prediction of cognitive decline after stroke; for example, hippocampal atrophy is a strong predictor for PSD outcome [12, 66]. Most acute stroke patients undergo CT brain imaging and, therefore, CT studies are representative of the whole clinical population. In everyday practice, CT is performed mainly to exclude intracerebral hemorrhage and certain stroke mimics (e.g., brain tumors), and can often show early signs of ischemia, as well as old stroke lesions. Additionally, the presence and extension of cerebral atrophy and white matter lesions can easily be seen on brain CT scans. Such neuroimaging characteristics may help predict subsequent cognitive impairment [67, 68].

MRI represents the most significant neuroimaging modality in PSD. If not contraindicated, MRI, rather than CT, is preferred for daily, routine clinical use as well as in research studies since it has higher specificity and sensitivity for detecting pathological substrates [66]. Neuroimaging standards with a generally accepted terminology allowing comparison of findings between different centers have been suggested (STandards for ReportIng Vascular changes on nEuroimaging, STRIVE) [69]. A large number of studies recognized MRI signs of cerebral SVD (lacunes, white matter hyperintensities, microbleeds, silent infarcts, white matter changes), as well as global cerebral atrophy and medial-temporal lobe atrophy as determinants and predictors of PSD. Vascular lesions associated with PSD are mainly found in subcortical brain areas, especially including sub-frontal and orbitofrontal white matter circuits. These lesions include single infarction in strategic areas such as the dominant thalamus or angular gyrus, deep areas of frontal lobe, and the left hemisphere, as well as brain infarcts in both hemispheres and volume-driven infarctions reaching a critical threshold of brain tissue loss or injury [70]. Additionally to size and location of the vascular lesion, the involvement of functional network fiber tracts, assessed by diffusion tensor imaging, may be crucial for cognitive impairment after stroke; its role is still being studied [71]. Although brain atrophy is a frequent finding in cerebral SVD, the pathophysiological mechanisms are not fully elucidated. In a recent study, Duering et al. [72] showed that cortical neurodegeneration following ischemia in the subcortical region probably represents pathophysiological mechanisms for cerebral atrophy in cerebrovascular disease; they showed that subcortical infarcts trigger focal thinning in connected cortical areas. Additionally, patients with mild VCI showed clear progressive gray matter atrophy in cortical (temporal and frontal) and subcortical (pons, caudate and cerebellum) regions after first-ever lacunar stroke, in contrast to patients without initial cognitive impairment [73].

Mild to moderate stroke patients with pre-existing white matter lesions are more vulnerable to cognitive impairment regardless of their new ischemic lesions [74]. Results from the SMART-MR study suggested that the interaction between brain atrophy and white matter hyperintensities or infarcts could aggravate cognitive decline [75]. There is evidence of the role of hippocampal mean diffusivity in the post-stroke cognitive state, above and beyond that of volume and connectivity of this structure [76].

Imaging of cerebral blood flow and cerebral glucose metabolism, by FDG-PET in particular, has been applied for several years in the differentiation between dementias [77]. However, functional and molecular imaging has a special role for research on the pathophysiology and the factors involved in development of PSD. Stroke and the deposition of amyloid in the cerebral cortex are both known risks for developing dementia [78, 79]. While animal models suggest that stroke-induced inflammation and amyloid deposition act synergetically [79], this relationship remains to be established in human stroke. Experimentally, large vessel infarcts or small striatal infarcts are larger in the presence of extracellular amyloid beta (A_β_) deposits, which are fundamental markers of AD. Patients with minor cerebral strokes and moderate AD lesions will develop the clinical manifestations of dementia. A reasonable question is whether the stroke was just a signal and not necessarily a player in the pathogenesis of cognitive deterioration. Additionaly, small striatal infarcts in the presence of high levels of amyloid in the brain exhibit a progression in infarct size over time with an enhanced degree of cognitive impairment, AD-type pathology, and neuroinflammation compared with striatal infarcts or high amyloid levels alone. Recently, it has been stated that stroke is a specific vascular risk factor for cognitive impairment [80]. There is an overlap between VCI risk factors (hypertension, diabetes, and atherosclerosis) and those for stroke, as well as AD risk factors. Stroke may alter the clinical expression of a given load of AD pathology, but more studies are needed for definitive clarification of the relationships between stroke, AD, and cognitive detoriation.

## Interventions

The best way to prevent PSD is to prevent stroke recurrence and stroke severity through optimal acute treatment and intensive secondary prevention. Intravenous and intra-arterial interventions (if indicated), treatment in the stroke unit including prevention of complications, and early rehabilitation are thought to limit the damage from the stroke lesion and improve outcome [81]. Secondary prevention includes medical interventions and lifestyle modification. To date, there is limited evidence for specific therapeutic strategies for preventing cognitive decline after stroke. However, aggressive treatment of vascular risk factors may potentially prevent PSD through prevention of the total vascular burden, including progression of vascular disease and vascular risk associated with dementia. An observational study showed reduced cognitive impairment in patients with appropriate vascular risk management post-stroke [82]; this included antiplatelet therapy, antihypertensive drugs and statins, or anticoagulants, if indicated. In addition, cognitive function can benefit from treatment of neuropsychiatric symptoms like depression, apathy, and anxiety, as well as cognitive training/stimulation [83].

In a pilot study, the relationship between amyloid deposition, microglia activation, and cognitive performance in stroke patients was investigated [84]. Preliminary results suggest that cortical amyloid deposition and post-stroke white matter inflammation contribute to PSD, indicating that various pathophysiological mechanisms are involved. If confirmed in larger trials, this finding might offer possibilities for clinical intervention to prevent post-stroke cognitive decline by modulation of inflammation or amyloid deposition. In addition, endothelin dysfunction with secondary neuroinflammation seen in acute non-atherothrombotic etiology stroke can be a potential target for drug treatment [85].

## Pharmacological interventions for prevention of PSD

### Blood pressure lowering

A review by Zanchetti et al. [86] of randomized controlled trials (RCTs) not limited to stroke patients suggests that blood pressure lowering may have some beneficial effects on cognitive decline. Hypertension treatment after stroke preserves cognition through prevention of recurrent stroke, but it is not yet clear whether it prevents cognitive decline through other mechanisms. Thus far, only four published RCTs restricted to patients with a previous stroke tested the effect of antihypertensive drugs on cognition as secondary outcome. The Perindopril Protection Against Recurrent Stroke study showed that long-term blood pressure lowering after stroke with perindopril was associated with reduced cognitive decline [87]. This effect was more pronounced in patients with recurrent stroke, suggesting a beneficial effect due to secondary stroke prevention. In contrast, telmisartan did not change cognition in the Prevention Regimen for Effectively Avoiding Second Strokes (PRoFESS) study [88]. Further, results of the Secondary Prevention of Small Subcortical Strokes (SPS3) trial showed no differences in cognitive function amongst groups according to blood pressure control or antiplatelet drug used [89]. One study, with cognition as the primary outcome, did not find any association between cognitive test performance and different blood pressure goals [90]. Similarly, candesartan did not improve cognitive function at 6 months when compared to placebo in the Scandinavian Candesartan Acute Stroke Trial (SCAST) [91].

### Statin treatment

Statin therapy in secondary stroke prevention has shown an effect on new vascular events but only marginally reduces the risk of stroke recurrence [92]. Statins lower LDL cholesterol and may have a beneficial effect on platelet function, endothelial activity, and inflammation [93]. No study has tested the effect of statins on cognition in patients with previous stroke. However, two large studies (HPS and PROSPER), which included patients with risk factors for, or a history of, vascular diseases, failed to show that either simvastatin or pravastatin affected cognition [94, 95].

### Other potential pharmacological interventions

Many therapeutic strategies have been developed for neuroprotection in acute stoke. Nitric oxide donors are thought to reduce infarct size and improve cerebral blood flow, but did not improve cognitive function post-stroke [96]. Acetylcholinesterase inhibitors and memantine may have a beneficial effect on cognition in patients with mild to moderate vascular dementia in general [97], but evidence for both general improvement are limited. Cerebrolysin had a beneficial effect on global outcome, but did not assess cognition, especially in stroke patients [98]. Selective serotonin reuptake inhibitors may be associated with overall recovery after stroke even in the absence of depression [99]. Fluoxetine is proposed to improve stroke recovery, and there is an ongoing trial that includes cognition as an outcome [100]. The dietary nucleotide citicoline may also improve recovery [101]. There are ongoing studies to explore the potential positive effect of phosphodiesterase-3, anti-inflammatory agents, BBB modulators, endothelin antagonist, flavonoids, immunosuppressive agents, peroxisome proliferator-activated receptor gamma antagonists, prostacylin, sympathomimetics, xanthine oxidase inhibitors, antidepressants, neurotrophic agents, gingko biloba extract Egb-761, and others [12, 102].

## Lifestyle interventions

### Multifactorial interventions

Most vascular risk factors are modifiable. Modifications of lifestyle factors include physical exercise, healthy diet, moderate alcohol consumption, and smoking cessation. Multimodal lifestyle interventions have been successful in changing the lifestyle habits of stroke survivors [103, 104]. Two randomized trials have been performed with multifactorial interventions including optimal medical treatment for secondary prevention in addition to physical activity, diet, weight control, smoking cessation, healthy diet with better glycemic control, and cognitive stimulation. One tested the effect of the multimodal risk factor intervention over 1 year on cognition, but no change in cognitive outcome was found [105]. However, significant beneficial effects were found on depression as the secondary outcome [106]. The recently published RCT by Matz et al. [107] showed that stroke patients receiving multiple, intensive life-style interventions did not differ in cognitive outcomes after 2 years compared to patients who received standard care; a small non-significant reduction in dysexecutive syndromes appearing in the intervention group was noted, but not significant. This finding indicates that studies for lifestyle interventions have to be performed in larger populations with neuropsychological functions as outcome measures, particularly the executive domains.

### Physical interventions

A systematic review including nine studies, published in 2012, showed that increased physical activity after stroke improves cognitive performance [108]. Exercise training is standard for cardiovascular disease management, and cardiac rehabilitation was shown to improve cognitive performance in several studies [109]. Sharing the same risk factors as stroke, brain rehabilitation including exercise might be a right strategy in future stroke care, supported by papers published over the past 2 years. A 6-month training model combining aerobic and resistance training, initiated at least 10 weeks post-stroke, resulted in improved cognitive function and reduction of patients meeting the threshold criteria for mild cognitive impairment [110]. A recently published paper by Moore et al. [111] showed that exercise therapy three times per week for 19 weeks improved cognitive function, increased regional blood flow in the medial temporal lobe, and prevented structural loss in the same region. An ongoing RCT, Move IT, aims to investigate whether a physical exercise program can prevent cognitive decline in the acute phase after transient ischemic attack or minor ischemic stroke [112].

### Cognitive training

Cognitive rehabilitation is a complex therapeutic intervention aiming to improve cognitive function after stroke; at present, the approached strategies are individual remediation therapy with a neuropsychologist, group-based training, and computer cognitive training [113]. Cognitive rehabilitation plays an integral role in stroke multidisciplinary rehabilitation and should be started shortly after stroke onset, with effectiveness both in the post-acute period and a few years after stroke [114]. However, there are insufficient data to conclude on the long lasting benefits in memory and executive function for post-stroke survivors [114, 115]. Moreover, data is missing regarding whether the possible improvements in neuropsychological tests are related to improvement in everyday functional activities [116].

### Non-invasive brain stimulation

Non-invasive brain stimulation techniques, including repetitive transcranial magnetic stimulation and transcranial direct current stimulation, have been reported to improve functional status of stroke patients through modulation of the excitability of cortical circuits [117, 118]. Nevertheless, most previous studies included a small number of participants and evaluated only the short-term effect. Therefore, there is limited evidence for recommendation of repetitive transcranial magnetic stimulation or transcranial direct current stimulation in the treatment of PSCI. Future studies are needed to elucidate appropriate stimulation sites, optimal parameters, and stimulation protocols to overcome individual differences such as skull shape or genetic polymorphisms [119].

### Actovegin

Natural biological products, such as actovegin, may have beneficial effects in the restorative phase of ischemia [120]. Elmilnger et al. [121] found protective and anti-oxidative effects of the hemodialysate actovegin on primary rat neurons in vitro. The efficacy of actovegin was investigated by Mikhaĭlova et al. [122] in the treatment of mild cognitive impairment post-stroke. The authors found a positive therapeutic effect, including increased speed of mental processes and reduced memory impairment as well as a positive impact on asthenic and depressive symptoms. In addition, the ARTEMIDA study explored whether this treatment has a disease-modifying effect on PSD [123]. Actovegin has been shown to have effects on some cellular processes in the aging brain and recent experimental studies revealed actovegin to play a role in neuroprotective mechanisms [124].

## Conclusions and further prospects

A variety of classifications, diagnostic criteria, and descriptive syndromes are used to define post-stroke cognitive problems, but a widely accepted and harmonized terminology is still missing. We propose the term PSD for any dementia following stroke that has a temporal relationship with stroke.

The best way to prevent PSD is optimal treatment to reduce stroke incidence and severity and, once stroke occurs, intensive prevention of early complications and long-term stroke recurrence. To date, there is limited evidence for specific therapeutic strategies for preventing cognitive decline after stroke. However, aggressive treatment of vascular risk factors, such as antiplatelet therapy, antihypertensives and statins, or anticoagulant therapy (if indicated), may potentially prevent PSD through inhibition of the total vascular burden, including progression of vascular disease and vascular risk associated with dementia. In addition, cognitive function can benefit from treatment of neuropsychiatric symptoms like depression, apathy, and anxiety, as well as cognitive training/stimulation.

In the future, it is necessary to introduce a uniform definition of cognitive impairment state following stroke, which should be widely accepted, leading to more accurate epidemiological results and a greater potential to compare and pool data. For this to be possible, all inclusion and exclusion criteria should be precisely defined, and in particular the time assessment of cognitive impairment following stroke. We propose that final diagnosis of PSD should be delayed to at least 6 months after the event.

One of the main strategies for easier identification of PSD is finding a precise biochemical marker. Since there is still no reliable marker, the search continues, with an enormous influence on future health economics and the development of preventative treatments and strategies. Therefore, multi-center studies on long-term cognitive outcomes in stroke patients should be given high priority.
